# Dietary carbohydrate intake and risks of overall and 21 site-specific cancers: a prospective cohort study

**DOI:** 10.3389/fnut.2025.1607358

**Published:** 2025-06-18

**Authors:** Shuhui Chen, Baojie Hua, Bin Liu, Le Wang, Qi Yuan, Yudan Yang, Xiaohui Sun, Ding Ye, Lingbin Du, Yingying Mao, Jiayu Li

**Affiliations:** ^1^Department of Epidemiology, School of Public Health, Zhejiang Chinese Medical University, Hangzhou, China; ^2^Department of Cancer Prevention, Zhejiang Cancer Hospital, Hangzhou Institute of Medicine (HIM), Chinese Academy of Sciences, Hangzhou, China

**Keywords:** dietary carbohydrate, sugar, diet, cancer, cohort study

## Abstract

**Background:**

Cancer is among the world’s top causes of death, and diet plays an important role in cancer risk. However, few studies have addressed a comprehensive atlas that details the connections between dietary carbohydrates and cancer risk.

**Methods:**

We conducted a large population-based prospective cohort research based on the UK Biobank including 194,388 participants. The Oxford WebQ, a web-based 24-h recall questionnaire, was used to collect dietary information of each study participant. Using the Cox proportional hazards model, we calculated the hazard ratios (HRs) with 95% confidence intervals (CIs) for the associations of energy-adjusted carbohydrates intake and the incidence of overall cancer as well as 21 site-specific cancers.

**Results:**

A total of 19,990 incidences of cancer (excluding non-melanoma skin cancer) were recorded with a median follow-up of 12.8 years. Energy-adjusted fiber was associated with a reduced risk of overall cancer [HR _per IQR increase_ (95% CI): 0.97 (0.96, 0.99); *P_FDR_*: 0.045] and esophageal [0.79 (0.68, 0.91); 0.024], colorectal [0.92 (0.87, 0.97); 0.025], lung [0.87 (0.81, 0.94); 0.014], and kidney cancer [0.85 (0.76, 0.94); 0.031]. Energy-adjusted free sugars were tied to a higher risk of lung [1.12 (1.05, 1.19); 0.024] and kidney cancer [1.15 (1.05, 1.26); 0.039], while non-free sugars were associated with a reduced risk of overall cancer [0.97 (0.95, 0.99); 0.031], colorectal [0.89 (0.84, 0.94); 0.006] and lung cancer [0.86 (0.79, 0.93); 0.014]. Finally, energy-adjusted sucrose was associated with an elevated risk of both lung cancer [1.10 (1.04, 1.17); 0.024] and non-Hodgkin lymphoma [1.15 (1.07, 1.23); 0.008].

**Conclusion:**

Increased consumption of dietary fiber and non-free sugars is associated with a reduced risk of certain cancers (e.g., overall cancer, esophageal, colorectal, lung, and kidney cancers), potentially due to their anti-inflammatory effects, short-chain fatty acid production, and other protective mechanisms. In contrast, higher intakes of free sugars and sucrose are associated with an elevated risk (e.g., lung, kidney cancer, and non-Hodgkin lymphoma), which may be attributed to inflammation and oxidative stress.

## Background

Cancer is among the world’s top causes of death, with rates of both incidence and mortality rising at an accelerated pace (1). In 2020, there were 19.3 million new cases of cancer worldwide, and roughly 10 million deaths from the illness (2), putting a heavy load on healthcare systems and economies almost everywhere. However, a study in the UK pointed that nearly 40% of cancer cases could potentially be avoided by addressing modifiable factors, such as diet (3). Given this substantial potential for cancer prevention, it is essential to look into the connection between dietary nutrients and cancer risk.

Carbohydrates are a primary source of energy in daily diets, supporting brain function and physical performance (4). Recent evidence suggests that total carbohydrate intake may affect cancer risk. For example, a meta-analysis revealed that high carbohydrate consumption was protective against esophageal cancer (5), while another study found that high carbohydrate intake was associated with an elevated risk of colorectal cancer (6). These conflicting findings suggest that the impact of total carbohydrate intake on cancer may vary by cancer site. Thus, more comprehensive investigation is required to evaluate the relationship between total carbohydrate and cancer risk.

Recent research suggests that understanding specific types of carbohydrates consumed, rather than total carbohydrate consumption, may be more effective for cancer prevention (7). The main components of carbohydrates include sugars, starches, and fiber (8), with their detailed classifications illustrated in Supplementary Figure S1. Previous studies have concentrated on isolated associations between individual types of carbohydrates and specific cancers. This approach has resulted in fragmented insights, with findings often lacking consistency. For instance, Meinhold et al. (9) and Jiao et al. (10) conducted separate prospective studies examining the impacts of sugars on the risk of pancreatic cancer. However, one study found that high sucrose intake was associated with increased cancer risk (9), whereas the other study found the opposite, with high fructose intake associated with increased cancer risk (10). Similarly, studies examining the impact of fiber on kidney cancer and bladder cancer also obtained inconsistent findings (11, 12). Therefore, to date, the relationships of different types of carbohydrates with various types of cancer remain unresolved, and there is a lack of a comprehensive atlas that details the comprehensive connections of different types of carbohydrates with cancer risk.

The UK Biobank (UKB) is a large-scale, prospective population cohort that has conducted dietary surveys on over 210,000 participants, providing a rich source of data on dietary habits, including information on more than 206 different foods (13). Using the UK Nutritional Database, carbohydrates have been categorized into three main groups (sugars, starch, and fiber) and further divided into up to 11 subtypes (14). This comprehensive dataset enables detailed analyses of dietary carbohydrate consumption and provides robust, high-quality evidence for understanding the role of carbohydrate intake in cancer risk. Given this unique resource, our study aims to fill the existing research gaps by examining the associations between total carbohydrate intake, various carbohydrate subtypes, and the incidence of overall cancer and 21 site-specific cancers in a large-scale prospective cohort. In addition, we conducted stratified analyses to explore potential modification effects by age and sex.

## Methods

### Design of the study and participants

We conducted a population-based prospective cohort study based on the UKB. From 2006 to 2010, 22 centers in the UK recruited more than 500,000 participants aged 37–73 years. Personal data, including as demographics, lifestyle variables, and health-related ailments, were gathered at baseline. North West Multi-center Research Ethics Committee approved the research project, and informed consent forms were signed by all participants (15).

**Figure 1:**
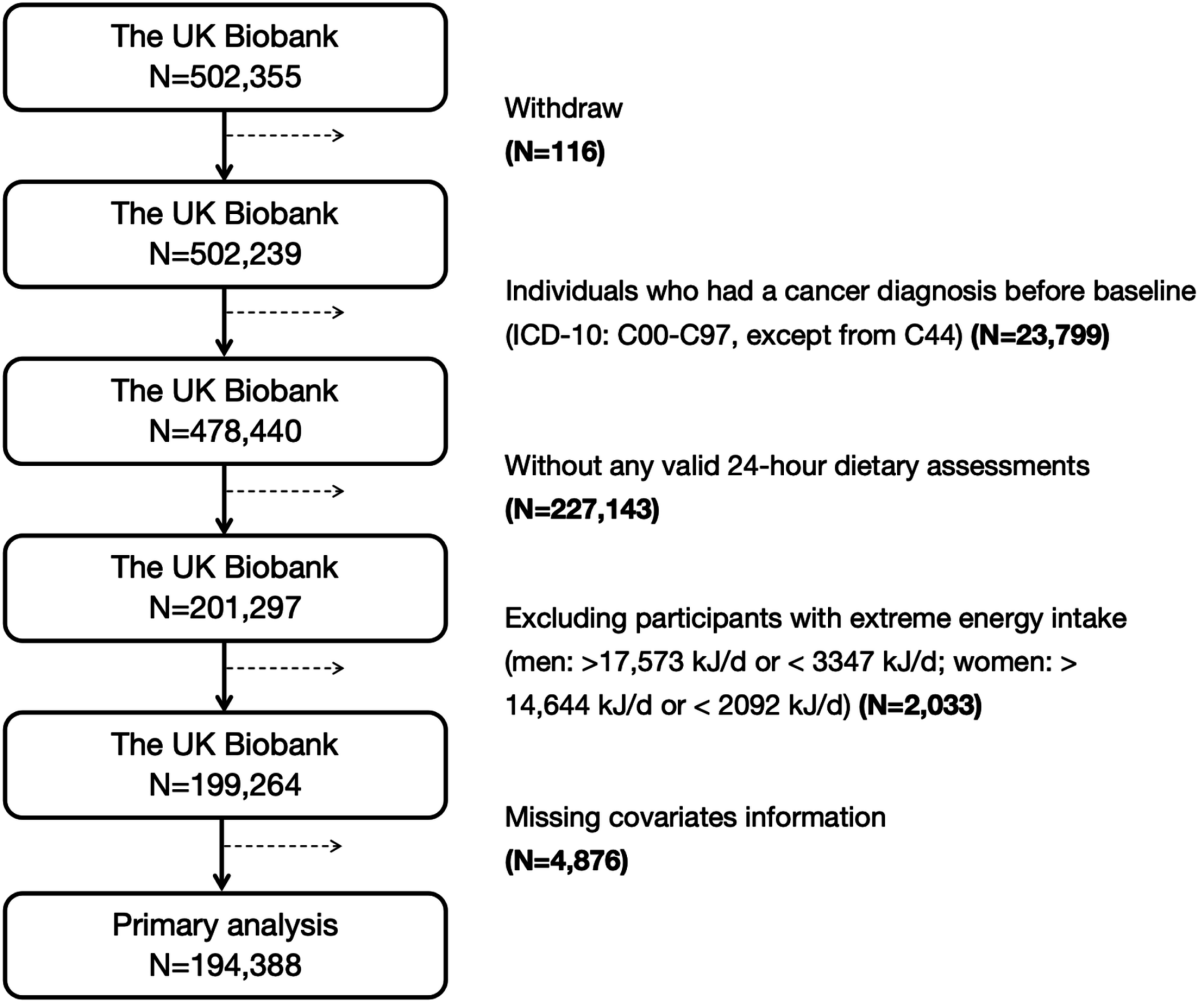
The inclusion and exclusion process of participants in this study. ICD-10, the International Classification of Diseases, tenth revision.

A total of 502,355 participants were included in this study. After excluding individuals who withdrew (*N* = 116), those who had any cancer diagnosis before baseline [International Classification of Diseases, Tenth Revision (ICD-10) codes: C00-C97, excluding C44] (*N* = 23,799), individuals without 24-h dietary assessment data (*N* = 227,143), those with extreme energy intake [men: >17,573 kJ/d or <3,347 kJ/d; women: >14,644 kJ/d or <2,092 kJ/d (16)] (*N* = 2,033), or those with missing covariates information (*N* = 4,876), a final sample of 194,388 participants were remained for the primary analyses. Among these participants, only 88,017 men were included in the prostate cancer analysis, and 106,371 women were included in the breast, corpus uteri, and ovarian cancer analyses (17). A flow chart of study participants inclusion and exclusion is present in Figure 1.

### Assessment of dietary carbohydrate

Dietary data for this study were collected using the Oxford WebQ, a web-based 24-h recall questionnaire, which captured information on up to 206 food items and 32 beverage items (13). Nutrient intake was estimated using the food composition tables from the UK Nutritional Database (14). Carbohydrates were categorized into three main types: sugars, starch, and fiber. Sugars were further divided into free sugars and non-free sugars, based on whether they were added during food processing or naturally present in whole foods. Additionally, sugars were classified by chemical structure into monosaccharides (e.g., glucose, fructose) and disaccharides (e.g., sucrose, lactose, maltose).

The dietary survey included up to five rounds of data collection. The first round was conducted between April 2009 and September 2010, during which approximately 210,000 participants were invited to complete the baseline Oxford WebQ questionnaire at the assessment centers. Participants who provided a valid email address were then invited to complete four additional online assessments between February 2011 and June 2022 (14). For the current analyses, when only a single 24-h dietary assessment was available, carbohydrate intake was calculated based on that single measurement. For participants with two or more assessments, intake was averaged to reduce measurement error and better reflect long-term dietary patterns (18).

We adjusted each carbohydrate type for total energy intake using the residual method (19). Specifically, we fitted a linear regression model with each carbohydrate type as the dependent variable and total energy intake as the independent variable. The resulting residuals capture the variation in carbohydrate intake that is independent of total energy intake. All carbohydrate variables used in the subsequent analyses are based on these energy-adjusted values to account for the effect of total energy intake on nutrient consumption.

### Assessment of outcome

Data on individuals’ cancer outcomes were obtained from the UK national cancer registry.^1^ Our study outcomes included overall cancer and site-specific cancers. Based on the World Health Organization’s (WHO) list of prevalent cancer types,^2^ we selected the most common cancer types for analysis. Following the approach of previous studies (20, 21), we included specific cancer types with more than 100 incident cases during the follow-up. Non-melanoma skin cancer was excluded from the overall cancer analysis due to incomplete registration and inconsistent recording practices, as it is common, typically non-fatal, and often managed outside major cancer registries (22–24). As a result, the final analyses encompassed overall cancer (excluding non-melanoma skin cancer) and 21 site-specific cancers (including head and neck, esophageal, stomach, colorectal, liver, gallbladder, pancreas, lung, melanoma of skin, mesothelioma, breast, corpus uteri, ovary, prostate, kidney, bladder, brain and central nervous system, thyroid, non-Hodgkin lymphoma, multiple myeloma, and leukaemia), with the corresponding diagnostic ICD-10 codes shown in Supplementary Table S1. The follow-up period was determined by measuring the time from the date of baseline recruitment to the first outcome diagnosis, death, loss to follow-up, or the end of follow-up (June 1, 2022), whichever occurred first.

### Covariates

According to previous studies, we selected covariates including age at recruitment, sex, ethnicity, Townsend Deprivation Index (TDI), education, smoking status, alcohol drinking status, body mass index (BMI), physical activity, energy-adjusted protein intake, and energy-adjusted fat intake (8, 25). TDI was used to gauge socioeconomic status, which integrates four indicators: household overpopulation, unemployment, and non-ownership of a vehicle or home. We classified participants into three tiers based on TDI tertiles, where tertile 1 (T1) group represents the least deprived population and tertile 3 (T3) group represents the most deprived population (26). Weight (in kilograms, kg) divided by height (in meters, m) squared yields the BMI (kg/m^2^). A person was considered to be physically active if he/she completed a weekly commitment of 150 min of moderate activity or 75 min of intense exercise, or if he/she engaged in vigorous exercise once a week or moderate exercise 5 days a week (27).

### Statistical analysis

R software version 4.4.1 was used for all statistical analyses. Categorical variables were reported as counts (percentages) and compared with Chi-square tests, whereas continuous variables were expressed as medians (interquartile ranges, IQRs) and compared using Mann–Whitney U tests. The relationships of energy-adjusted carbohydrates with the incidence of overall cancer and 21 site-specific cancers were examined using the Cox proportional hazards model. Model 1 was adjusted for age at recruitment and sex (not adjusted in prostate, breast, corpus uteri, and ovary cancer analyses) and model 2 was further adjusted for ethnicity, TDI, education, smoking status, alcohol drinking status, BMI, physical activity, energy-adjusted protein intake, and energy-adjusted fat intake. To evaluate the potential for multicollinearity, we calculated the variance inflation factors (VIF) for all variables in each carbohydrate analysis. These results indicated that all VIF values were below 5, suggesting no substantial multicollinearity. The false discovery rate (FDR) approach was utilized to correct the *p*-values of the associations of each IQR increase in carbohydrate intake with the risk of overall cancer and 21 site-specific cancers. For the statistically significant associations of carbohydrates with cancer that were noted in the above analyses (FDR-*p* < 0.05), further stratified analyses were performed by age at recruitment (<60 or ≥60) and sex (male or female). The heterogeneity between the different strata was evaluated using Cochran’s Q tests (*meta* R package). Sensitivity analyses in this study included: (1) eliminating those who were diagnosed with any cancer (ICD-10 codes: C00-C97, excluding C44) within the first 2 years of the follow-up; (2) limiting participants to those with typical diet throughout 24-h dietary evaluations; (3) executing multiple imputations for missing covariates using the *mice* R package, and reanalyzing the relationships of energy-adjusted carbohydrates with the incidence of overall cancer and various types of cancers; (4) additionally adjusting for prevalent hypertension (ICD-10 codes: I10-I13, I15), diabetes (ICD-10 codes: E10-E14), and dyslipidemia (ICD-10 code: E78); (5) additionally adjusting for overall fruit, vegetable, and processed meats intake.

## Results

### Baseline characteristics

A total of 19,990 incidences of cancer were recorded with a median follow-up of 12.8 years, with 10,530 (52.7%) occurring in males and 9,460 (47.3%) in females. Table 1 shows the baseline characteristics of the study participants. Compared to those without cancer, cancer cases were more likely to be men and older, predominantly White, and from more affluent backgrounds (with a greater proportion of “Low TDI” and “Medium TDI” and lower proportions of “High TDI”). However, they exhibited lower educational attainment, with a higher proportion holding vocational qualifications and a lower proportion with any school degree or higher degree. Additionally, cancer cases were more prone to smoking and alcohol use, had a higher BMI, and were physically inactive.

**Table 1:** Baseline characteristics of the study population.

Characteristics	Overall	Incident cancer events^a^	No cancer participants
Number	194,388	19,990	174,398
Age at recruitment (years), median (IQR)	57.00 (50.00, 62.00)	61.00 (55.00, 65.00)	56.00 (49.00, 62.00)
Sex, *N* (%)			
Female	106,371 (54.7)	9,460 (47.3)	96,911 (55.6)
Male	88,017 (45.3)	10,530 (52.7)	77,487 (44.4)
Ethnicity, *N* (%)			
White	185,957 (95.7)	19,392 (97.0)	166,565 (95.5)
Others	8,431 (4.3)	598 (3.0)	7,833 (4.5)
TDI, *N* (%)			
Low	69,784 (35.9)	7,406 (37.0)	62,378 (35.8)
Medium	66,716 (34.3)	6,975 (34.9)	59,741 (34.3)
High	57,888 (29.8)	5,609 (28.1)	52,279 (30.0)
Education, *N* (%)^b^			
Vocational qualification	10,560 (5.4)	1,271 (6.4)	9,289 (5.3)
Any school degree	74,245 (38.2)	7,134 (35.7)	67,111 (38.5)
Higher degree	93,766 (48.2)	9,393 (47.0)	84,373 (48.4)
None of the preceding groups	15,817 (8.1)	2,192 (11.0)	13,625 (7.8)
Smoking status, *N* (%)			
Never	110,387 (56.8)	9,944 (49.7)	100,443 (57.6)
Previous	68,803 (35.4)	8,207 (41.1)	60,596 (34.7)
Current	15,198 (7.8)	1,839 (9.2)	13,359 (7.7)
Alcohol drinking status, *N* (%)			
Never	6,074 (3.1)	560 (2.8)	5,514 (3.2)
Previous	5,771 (3.0)	637 (3.2)	5,134 (2.9)
Current	182,543 (93.9)	18,793 (94.0)	163,750 (93.9)
BMI (kg/m^2^), *N* (%)			
<25	73,256 (37.7)	6,561 (32.8)	66,695 (38.2)
25–29.9	80,777 (41.6)	8,844 (44.2)	71,933 (41.2)
≥30	40,355 (20.8)	4,585 (22.9)	35,770 (20.5)
Physical activity, *N* (%)			
No	38,530 (19.8)	4,151 (20.8)	34,379 (19.7)
Yes	155,858 (80.2)	15,839 (79.2)	140,019 (80.3)

BMI, body mass index; ICD-10, the International Classification of Diseases, tenth revision; IQR, interquartile range; TDI, Thomson deprivation index.

a Incident cancer events refer to any cancers that occurred during the follow-up period (ICD-10 codes: C00-C97, excluding C44).

b Vocational qualification (NVQ, HND, HNC, or equivalent), Any school degree (A level, AS level, O level, GCSE, CSE, or equivalent), Higher degree (college, university, or professional degree or qualification).

In terms of dietary intake, compared to participant without cancer, cancer cases consumed more energy and energy-adjusted lactose, but fewer energy-adjusted total carbohydrates, energy-adjusted starch and energy-adjusted other sugars (all *p* < 0.05), more details are shown in Supplementary Table S2.

### Primary analyses

**Figure 2:**
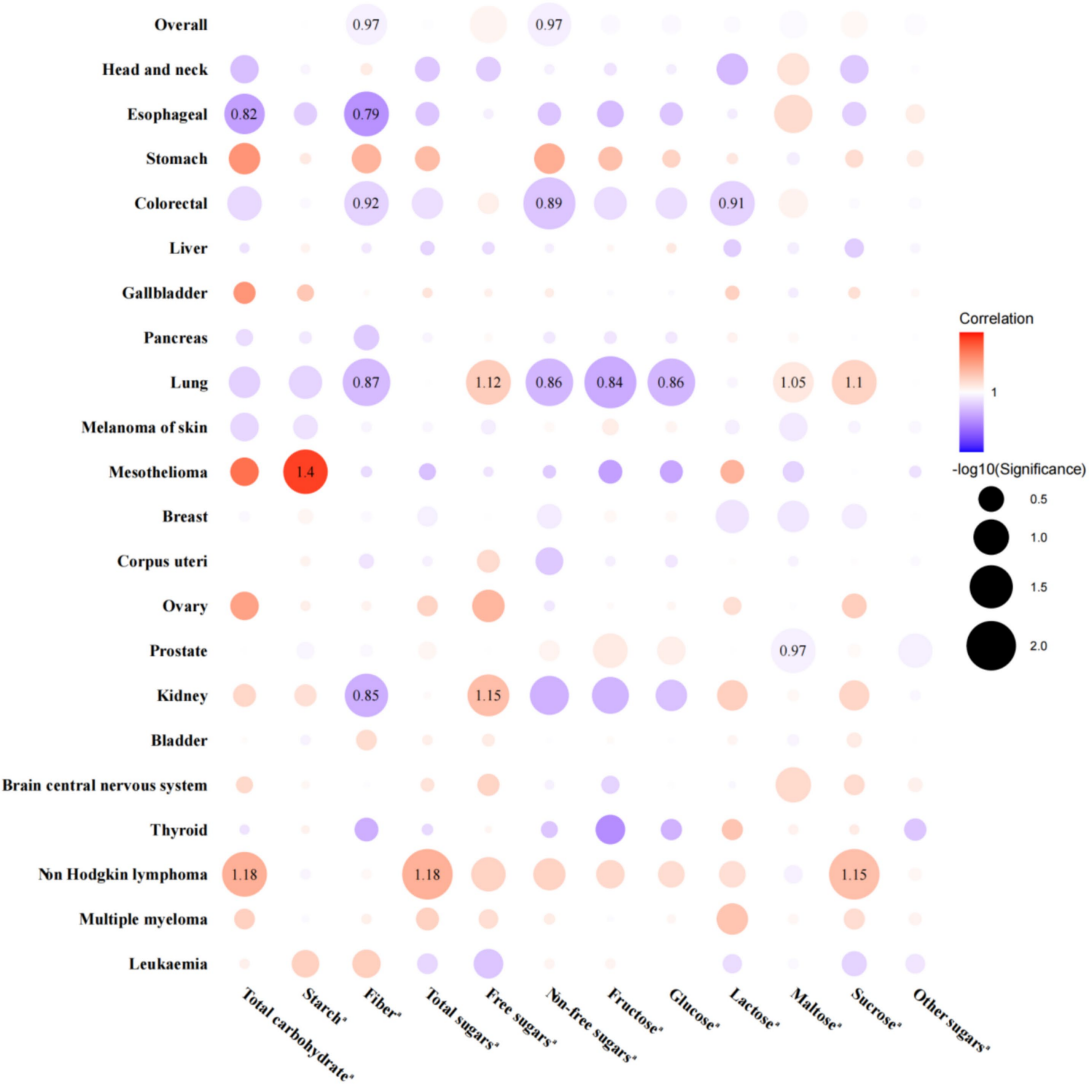
Associations between dietary carbohydrates per interquartile range increase and the risk of overall and site-specific cancers, with hazard ratios displayed only for FDR-adjusted significant associations. ^a^All carbohydrates were adjusted for total energy intake. Models were adjusted for age at recruitment, sex (not adjusted in prostate, breast, corpus uteri, and ovary cancer analyses), ethnicity, TDI, education, smoking status, alcohol drinking status, BMI, physical activity, energy-adjusted protein intake, and energy-adjusted fat intake. Red point indicates a positive association between carbohydrates and cancer risk, with darker shades representing stronger associations. Blue point indicates a negative association between carbohydrates and cancer risk, with darker shades also representing stronger associations. BMI, body mass index; FDR, false discovery rate; HR, hazard ratio; TDI, Thomson deprivation index.

Regarding the relationships between energy-adjusted carbohydrates per IQR increase and the risk of cancer at overall and 21 site-specific cancers, 54 associations were statistically significant, and 21 remained significant after FDR adjustment (Figure 2). To be specific, the energy-adjusted total carbohydrates was linked to a reduced risk of esophageal cancer [HR _per IQR increase_ (95% CI): 0.82 (0.71, 0.94); *P_FDR_*: 0.049] but an elevated risk of non-Hodgkin lymphoma [HR _per IQR increase_ (95% CI): 1.18 (1.07, 1.31); *P_FDR_*: 0.024]. Energy-adjusted starch was associated with a higher risk of mesothelioma [HR _per IQR increase_ (95% CI): 1.40 (1.14, 1.72); *P_FDR_*: 0.025]. Meanwhile, energy-adjusted fiber was associated with a reduced risk of overall cancer [HR _per IQR increase_ (95% CI): 0.97 (0.96, 0.99); *P_FDR_*: 0.045] and esophageal [HR _per IQR increase_ (95% CI): 0.79 (0.68, 0.91); *P_FDR_*: 0.024], colorectal [HR _per IQR increase_ (95% CI): 0.92 (0.87, 0.97); *P_FDR_*: 0.025], lung [HR _per IQR increase_ (95% CI): 0.87 (0.81, 0.94); *P_FDR_*: 0.014], and kidney cancer [HR _per IQR increase_ (95% CI): 0.85 (0.76, 0.94); *P_FDR_*: 0.031]. Energy-adjusted total sugars were linked to an increased risk of non-Hodgkin lymphoma [HR _per IQR increase_ (95% CI): 1.18 (1.09, 1.29); *P_FDR_*: 0.008]. When we delved deeper into the categories of sugar, we found that energy-adjusted free sugars were tied to a higher risk of lung [HR _per IQR increase_ (95% CI): 1.12 (1.05, 1.19); *P_FDR_*: 0.024] and kidney cancer [HR _per IQR increase_ (95% CI): 1.15 (1.05, 1.26); *P_FDR_*: 0.039], and non-free sugars appeared to offer a protective effect, associating with a reduced risk of overall cancer [HR _per IQR increase_ (95% CI): 0.97 (0.95, 0.99); *P_FDR_*: 0.031] as well as specific cancers, including colorectal [HR _per IQR increase_ (95% CI): 0.89 (0.84, 0.94); *P_FDR_*: 0.006] and lung cancer [HR _per IQR increase_ (95% CI): 0.86 (0.79, 0.93); *P_FDR_*: 0.014]. Additionally, energy-adjusted fructose [HR _per IQR increase_ (95% CI): 0.84 (0.77, 0.91); *P_FDR_*: 0.006] and glucose [HR _per IQR increase_ (95% CI): 0.86 (0.79, 0.94); *P_FDR_*: 0.014] intake corresponded to a lower risk of lung cancer. Energy-adjusted lactose was linked to a reduced risk of colorectal cancer [HR _per IQR increase_ (95% CI): 0.91 (0.87, 0.96); *P_FDR_*: 0.024]. On the flip side, energy-adjusted maltose intake was related to an increased risk of lung cancer [HR _per IQR increase_ (95% CI): 1.05 (1.02, 1.09); *P_FDR_*: 0.048] but a slightly reduced risk of prostate cancer [HR _per IQR increase_ (95% CI): 0.97 (0.96, 0.99); *P_FDR_*: 0.025]. Finally, energy-adjusted sucrose was associated with an elevated risk of both lung cancer [HR _per IQR increase_ (95% CI): 1.10 (1.04, 1.17); *P_FDR_*: 0.024] and non-Hodgkin lymphoma [HR _per IQR increase_ (95% CI): 1.15 (1.07, 1.23); *P_FDR_*: 0.008]. Detailed HRs with 95% CIs for the associations across quartiles are presented in Supplementary Table S3.

### Stratified analyses and sensitivity analyses

**Figure 3:**
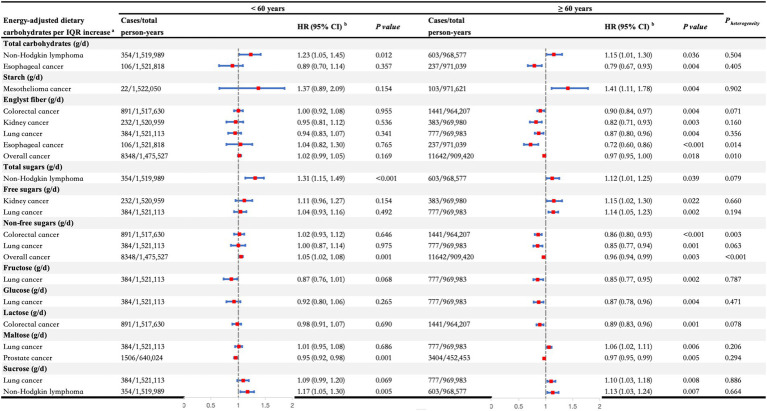
Stratified analyses of the associations between dietary carbohydrates per interquartile range increase and the risk of overall cancer and different types of cancers by age at recruitment. ^a^All carbohydrates were adjusted for total energy intake, and the significant associations between dietary carbohydrates and specific cancers observed in the main analyses were further analyzed in the stratified analyses. ^b^Adjusted for sex, ethnicity, TDI, education, smoking status, alcohol drinking status, BMI, physical activity, energy-adjusted protein intake, and energy-adjusted fat intake. BMI, body mass index; CI, confidence interval; HR, hazard ratio; IQR, interquartile range; TDI, Thomson deprivation index.

**Figure 4:**
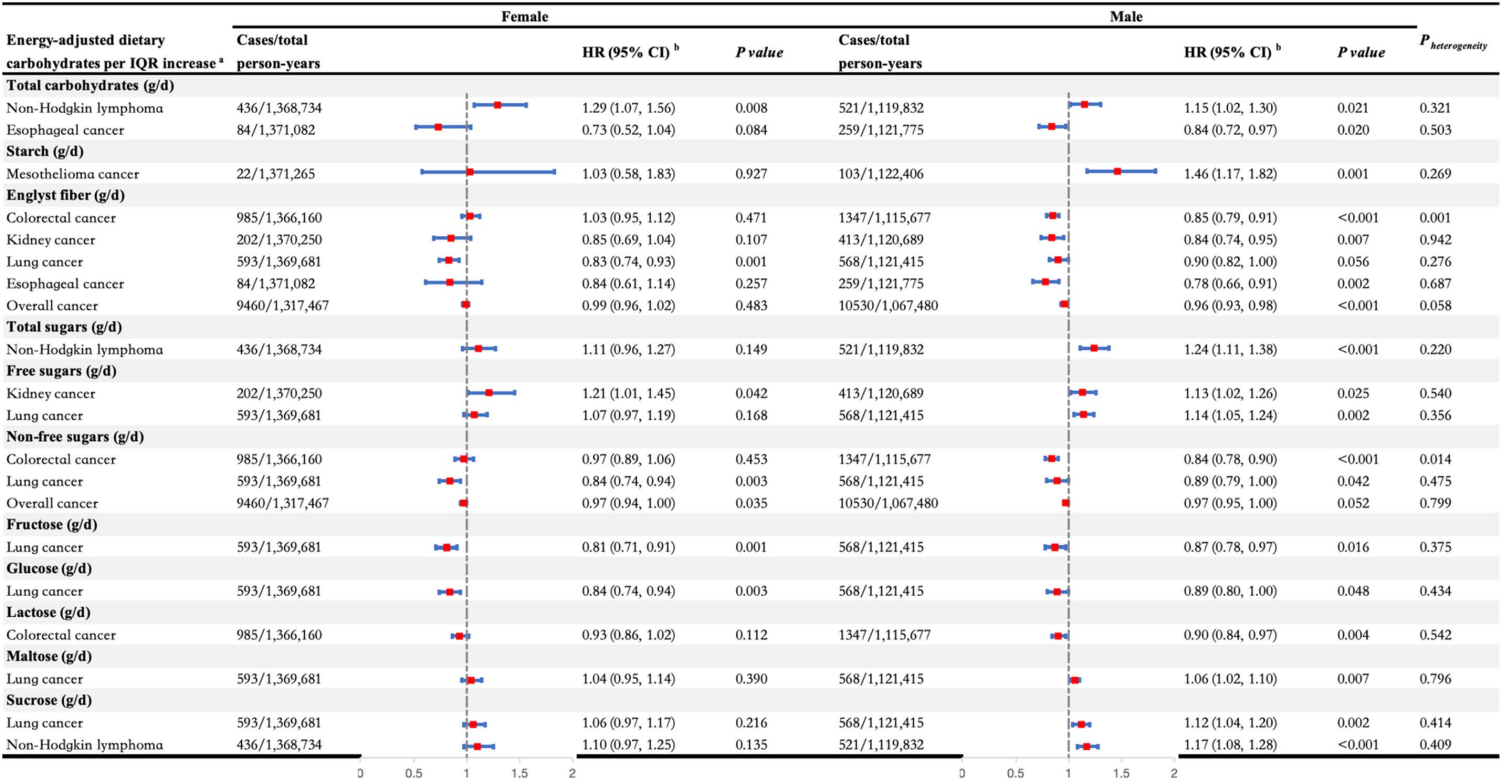
Stratified analyses of the associations between dietary carbohydrates per interquartile range increase and the risk of overall cancer and different types of cancers by sex. ^a^All carbohydrates were adjusted for total energy intake, and the significant associations between dietary carbohydrates and specific cancers observed in the main analyses were further analyzed in the stratified analyses. ^b^Adjusted for age at recruitment, ethnicity, TDI, education, smoking status, alcohol drinking status, BMI, physical activity, energy-adjusted protein intake, and energy-adjusted fat intake. BMI, body mass index; CI, confidence interval; HR, hazard ratio; IQR, interquartile range; TDI, Thomson deprivation index.

In the secondary analyses, we conducted stratified analyses on the significant associations identified in the primary analyses (Figures 3, 4). We observed heterogeneity in the associations between fiber and non-free sugars with overall cancer and gastrointestinal cancer across different age and sex strata (all *P _heterogeneity_* < 0.05). Specifically, for fiber, the protective effect of increased intake against overall cancer was significant only in individuals over the age of 60, with estimates of HR _per IQR increase_ (95% CI): 1.02 (0.99, 1.05) for those under 60 years and HR _per IQR increase_ (95% CI): 0.97 (0.95, 1.00) for those 60 years and older (*P _heterogeneity_* = 0.010). Similarly, the protective effect against esophageal cancer was significant only in individuals over 60, showing HR _per IQR increase_ (95% CI): 1.04 (0.82, 1.30) for those under 60 years versus HR _per IQR increase_ (95% CI): 0.72 (0.60, 0.86) for those 60 years and older (*P _heterogeneity_* = 0.014). In addition, the inverse association between fiber intake and colorectal cancer risk was considerable only in men, with estimates of HR _per IQR increase_ (95% CI): 1.03 (0.95, 1.12) for females and HR _per IQR increase_ (95% CI): 0.85 (0.79, 0.91) for males (*P _heterogeneity_* = 0.001). For non-free sugars, increased consumption was linked to a decreased risk of colorectal cancer, but this effect was significant only in individuals over 60, with estimates of HR _per IQR increase_ (95% CI): 1.02 (0.93, 1.12) for those under 60 years and HR _per IQR increase_ (95% CI): 0.86 (0.80, 0.93) for those 60 years and older (*P _heterogeneity_* = 0.003). This association was significant in males, showing HR _per IQR increase_ (95% CI): 0.97 (0.89, 1.06) for females and HR _per IQR increase_ (95% CI): 0.84 (0.78, 0.90) for males (*P*_heterogeneity_ = 0.014). Furthermore, higher non-free sugar intake was associated with a lower overall cancer risk in those over 60, while it increased the risk in those under 60, with estimates of HR _per IQR increase_ (95% CI): 1.05 (1.02, 1.08) for those under 60 years and HR _per IQR increase_ (95% CI): 0.96 (0.94, 0.99) for those 60 years and older (*P _heterogeneity_* < 0.001).

In sensitivity analysis, the association between carbohydrates and the risk of overall cancer and the 21 site-specific cancers identified in the main analyses did not significantly alter after eliminating those who were diagnosed with any cancer within the first 2 years of the follow-up, executing multiple imputations for missing covariates or additionally adjusted for prevalent diseases (hypertension, diabetes, and dyslipidemia). When the analyses were further restricted to participants with typical diets during the 24-h dietary assessments, the positive association between starch intake and mesothelioma risk persisted with a similar effect size, although the *p* value approached borderline statistical significance. Furthermore, after additionally adjusting for overall fruit, vegetable, and processed meats intake, the inverse associations between fiber, non-free sugars, and overall cancer risk were no longer statistically significant; however, their direction and magnitude remained largely consistent. Importantly, the associations for other significant carbohydrate-cancer pairs remained stable, further reinforcing the robustness of our main findings (Supplementary Table S4).

## Discussion

In this investigation, we explored the relationship between carbohydrates and the risk of both overall cancer and 21 site-specific cancers in 194,388 participants from the UKB. In two or more distinct cancer sites, larger intakes of dietary fiber and non-free sugars were consistently associated with a decreased risk, while larger intakes of free sugar and sucrose were consistently associated with an increased risk.

In light of our findings, higher intake of fiber was linked to a lower risk of overall cancer, and particular types including esophageal, colorectal, lung, and kidney cancers. Previous research has demonstrated the protective effect of fiber against several cancers (28). A prospective study investigating the link between fiber intake from various sources and cancer risk indicated that consuming fiber was linked to a lower incidence of overall cancer, esophageal, lung, and kidney cancer (11), which is aligning with our observations. Previous meta-analyses also found consistent associations between higher fiber intake and reduced risks of esophageal (29), renal cell cancers (30) and colorectal cancer (31). Compared to other cancers, the association between fiber intake and colorectal cancer is the most commonly reported. One important mechanism is that higher consumption of fiber enhances the production of butyrate by gut microbiota. This short-chain fatty acid is essential for causing apoptosis and preventing the development of cancer cells, thereby potentially lowering colorectal cancer risk. Moreover, butyrate also exerts anti-inflammatory effects, further reinforcing its protective role in colorectal cancer prevention (32). In addition, fiber in the gut can increase stool bulk, reduce colonic transit time, influence prebiotic effects, and regulate bile acid concentrations in the stool, all of which are suggested to play a role in colorectal carcinogenesis (33). The main dietary sources of fiber are whole grains, vegetables, and fruits, which also contain a wealth of protective minerals and vitamins against colorectal cancer. Interestingly, our study also found that non-free sugars were associated with a decreased risk of overall cancer, as well as colorectal and lung cancers. Non-free sugars are also abundant in fruits and vegetables, suggesting that fiber, non-free sugars, and protective minerals and vitamins may have a synergistic protective effect against colorectal cancer. Additionally, our study revealed that non-free sugars were linked to a decreased risk of overall cancer and lung cancer. Previous epidemiological evidence supports the notion that fruits and/or vegetables lower the risk of overall cancer and various types of cancer, including lung, colorectal, breast cancers, as well as prostate cancer (34, 35). However, current research on the relationship between non-free sugars and cancer, along with the underlying mechanisms, is still limited, warranting further investigation.

In contrast to non-free sugars, our findings indicate that consuming free sugars is associated with an increased risk of lung and kidney cancer. According to the WHO, sugar-sweetened beverages (SSBs) are defined as all beverages containing free sugars (36), which are the primary dietary source of free sugars (37). Several studies have specifically linked SSB consumption to a higher risk of kidney cancer (38, 39), while evidence for lung cancer remains limited. The potential carcinogenic mechanisms of free sugars may involve multiple pathways. First, excessive free sugar intake can lead to obesity, a well-established risk factor for cancer (37). Obesity is associated with chronic inflammation, characterized by elevated levels of inflammatory cytokines, which can promote cancer development through their pro-inflammatory and pro-tumorigenic effects (40). Additionally, frequent consumption of free sugars can cause rapid postprandial blood glucose spikes, increasing oxidative stress and promoting the formation of DNA-damaging reactive oxygen species (41), potentially contributing to the initiation and progression of cancer. Moreover, free sugars are often associated with lower-quality diets and reduced micronutrient intake (42), which may further compromise immune function and increase susceptibility to cancer (43).

Our research also suggests that higher sucrose intake may be associated with an increased risk of lung cancer. This observation aligns with a prior case–control study that identified a similar association between sucrose consumption and lung cancer risk (44). In parallel, animal studies using Lewis lung carcinoma models have demonstrated that high-sucrose diets can promote tumor growth and metastasis, potentially through an imbalance characterized by increased pro-angiogenic factors and decreased anti-angiogenic factors (45). Additionally, in mouse breast cancer models, sucrose-rich diets have been found to upregulate 12-lipoxygenase (12-LOX) and its arachidonic acid metabolite 12-hydroxy-5Z,8Z,10E,14Z-eicosatetraenoic acid (12-HETE), which have been linked to increased cancer cell invasiveness and metastatic potential (46). Collectively, these findings suggest that the pro-tumor effects of sucrose may involve both angiogenic and inflammatory pathways, underscoring the need for further research to clarify these mechanisms in the context of other cancers, such as non-Hodgkin lymphoma.

Evidence from our study suggests that a potential inverse association between maltose intake and prostate cancer risk, but a positive association with lung cancer risk. Previous studies examining the relationship between maltose intake and cancer risk have also produced mixed results, with one case–control study from Iran reporting a positive association between maltose intake and colon cancer risk (47), while a cross-sectional study from Japan found no such relationship (48). Additionally, we observed that higher intakes of glucose and fructose were associated with a lower risk of lung cancer, while greater lactose consumption was linked to a reduced incidence of colorectal cancer. Review evidence on the relationship between dietary sugars with different chemical structures and various cancer risks remains unclear, with some studies yielding conflicting findings (49), underscoring the need for further research to clarify these associations.

We observed that the inverse association between dietary fiber, non-free sugars, and colorectal cancer risk was significant only in men, while the associations in women were not statistically significant. This sex-specific difference may be influenced by variations in gut microbiota composition and hormonal factors. Experimental study has shown that male mice exhibit more pronounced shifts in gut microbiota composition in response to fiber-rich diets compared to female mice, particularly marked by an increase in fiber-fermenting bacteria such as *Proteus* and *Lactobacillus* (50). These bacteria produce short-chain fatty acids (51), which inhibit NF-κB activation and reduce pro-inflammatory cytokine expression (52), thereby potentially slowing or preventing tumor progression. This enhanced microbial capacity for fiber metabolism in males may contribute to the stronger protective effects observed in our study. In contrast, higher estrogen levels in women may obscure the protective effects of dietary fiber. Estrogen can activate estrogen receptor beta (ERβ/ESR2) in the colon, promoting apoptosis and reducing polyp formation, thereby inhibiting the early stages of colorectal carcinogenesis through mechanisms that might overlap with those of dietary fiber (53). This natural hormonal protection provided by estrogens may partially obscure the additional benefits of dietary fiber and non-free sugars in women, potentially explaining the observed sex-specific differences.

Additionally, the observed age-related heterogeneity in the effects of fiber and non-free sugars may reflect differences in dietary habits and physiological responses associated with aging. Older adults often have better overall diet quality (54) and longer cumulative exposure to dietary fiber and non-free sugars, which can contribute to maintaining gut microbiota balance and reducing chronic inflammation, thereby potentially lowering cancer risk. However, aging is also accompanied by immune senescence and chronic low-grade inflammation, partly driven by the accumulation of senescent cells and age-related gut microbiota dysbiosis (55, 56). These age-related changes can impair intestinal barrier function and increase systemic inflammation, creating a more vulnerable physiological environment. In this context, a diet rich in fiber or non-free sugars may help mitigate these adverse effects by promoting beneficial gut bacteria and reducing inflammation, potentially contributing to a stronger inverse association with overall and colorectal cancer risk in older adults compared to their younger counterparts.

The most recent WCRF/AICR recommendations emphasize that dietary and lifestyle patterns should prioritize fruit and vegetable consumption, as well as fiber-containing foods for overall cancer prevention (57). Our current research findings support this, showing that energy-adjusted fiber intake is associated with a reduced risk of overall cancer. Additionally, non-free sugars, with fruits being an important source, were linked to a lower risk of overall cancer. However, the WCRF/AICR recommendations for breast and colorectal cancer suggest limiting SSBs, which are a major source of free sugars. In our study, we did not find a significant association between free sugars and breast or colorectal cancer. Instead, we observed a link between free sugars and an increased risk of lung and kidney cancers. This conclusion requires further validation in larger cohorts with diverse dietary characteristics.

There are several advantages to this study. First, it used a design of a large-scale prospective cohort study, providing stronger evidence for causal associations. Moreover, it comprehensively assessed the relationships between various types of carbohydrates and multiple cancer types, thus allowing for a more thorough exploration of diet influences on cancer risk. However, this study also has some limitations. First, although a prospective study design was employed, it remains an observational study, and therefore, its causal relationships need further validation through approaches such as randomized controlled trials. Second, self-reported dietary data are vulnerable to various information biases, including recall bias, where participants may inaccurately remember their food intake, especially for infrequently consumed items (e.g., snacks); reporting bias, as individuals with obesity or chronic diseases might systematically misreport their energy intake; and errors in portion size measurement. Additionally, 24-h recall may not fully capture habitual intake. These biases can introduce a risk of misclassification, potentially weakening or strengthening the observed associations between exposure and outcome. In the current study, to address these biases, we excluded extreme energy reporters during participant selection. To estimate exposure factors, we aggregated multiple assessments to better approximate habitual intake. During data analysis, we conducted sensitivity analyses by restricting the population to those whose 24-h dietary recalls aligned with their usual eating habits, ensuring that the results remained consistent with the main analysis. In future studies, incorporating more refined dietary assessments, alongside objective biomarkers, may help further reduce these biases and improve the accuracy of dietary exposure estimates. Third, participants may have experienced changes in their dietary habits during the follow-up period; however, the UKB did not assess dietary intake during this time. As a result, this could introduce bias into the estimates of the relationship between carbohydrates and the outcomes in this study. Fourthly, the UKB cohort is subject to a “healthy volunteer” selection bias, which may have implications for external validity. Specifically, individuals with severe health conditions or lower socioeconomic status are less likely to participate, leading to representativeness issues in the study population. If there are significant differences in the distribution of exposure factors and other important confounders between participants in the UKB and the general population in the UK, it could potentially lead to inaccurate estimates of true disease risks and limit the generalizability of the conclusions. Fifthly, the study participants were predominantly White British. Due to significant dietary cultural differences among various regions, the types and amounts of carbohydrate intake vary. For example, in Mediterranean diets, whole grain bread is predominant at every meal. In contrast, East Asian diets are characterized by high rice consumption, which serves as the primary source of carbohydrates. Additionally, there are significant differences in recommended carbohydrate intake across different countries, which may also influence people’s choices regarding daily carbohydrate consumption and types. For instance, Canada has the most lenient guideline for added sugars (“maximum intake ≤25% of total energy”), while the strictest recommendations come from the UK (“free sugars: 5% of total energy”) (58). In the future, it will be necessary to further validate these conclusions in populations with diverse dietary features. Lastly, this study may also have residual confounding that was not adjusted for, such as glycemic index (data unavailable).

## Conclusion

Increased consumption of dietary fiber and non-free sugars is associated with a reduced risk of certain cancers (e.g., overall cancer, esophageal, colorectal, lung, and kidney cancers), potentially due to their anti-inflammatory effects, short-chain fatty acid production, and other protective mechanisms. In contrast, higher intakes of free sugars and sucrose are associated with an elevated risk (e.g., lung, kidney cancer, and non-Hodgkin lymphoma), which may be attributed to inflammation and oxidative stress.

## Data Availability

Publicly available datasets were analyzed in this study. This data can be found at: https://www.ukbiobank.ac.uk/.

## Footnotes

^1^ https://biobank.ndph.ox.ac.uk/ukb/label.cgi?id=100092

^2^ https://gco.iarc.who.int
