# Supplementary material for: Dietary carbohydrate intake and risks of overall and 21 site-specific cancers: a prospective cohort study
Source: Front Nutr. 2025 Jun 18;12:1607358. doi: 10.3389/fnut.2025.1607358 (PMC12213366; doi:10.3389/fnut.2025.1607358)
Supplement: Supplementary file 1 [file Table_1.docx]

**Supplementary files**

**Title: Dietary Carbohydrate Intake and Risks of Overall and 21 Site-Specific Cancers: A Prospective Cohort Study**

**Authors:** Shuhui Chen^1^, Baojie Hua^1^, Bin Liu^1^, Le Wang^2^, Qi Yuan^1^, Yudan Yang^1^, Xiaohui Sun^1^, Ding Ye^1^, Lingbin Du^2^, Yingying Mao^1,^*, Jiayu Li^1,^*

| **Content** | **Page** |
| --- | --- |
| **Figure S1.** Detailed Classification of Carbohydrates | P2 |
| **Table S1.** ICD-10 codes of cancer outcomes in this study | P3 |
| **Table S2.** Characteristics of energy-adjusted carbohydrates intake in the study population | P4 |
| **Table S3.** Hazard ratios for the associations between dietary carbohydrates across quartiles and the risk of overall cancer and various types of cancers | P5-7 |
| **Table S4.** Sensitivity analyses on the associations between dietary carbohydrates and the risk of overall cancer and various types of cancers | P8-12 |


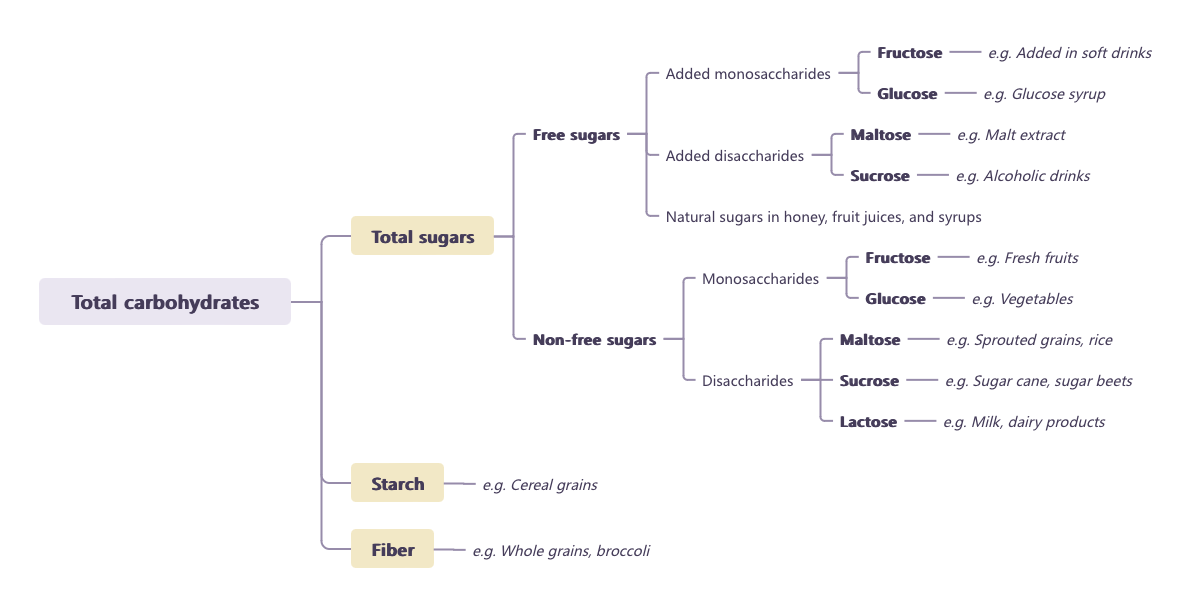


**Figure S1.** Detailed Classification of Carbohydrates.

| **Table S1. ICD-10 codes of cancer outcomes in this study** | |
| --- | --- |
| **Cancer sites** | **ICD-10 codes** |
| Overall | C00-C97 excluding non-melanoma skin cancer(C44) |
| Head and neck | C00-C14 |
| Esophageal | C15 |
| Stomach | C16 |
| Colorectal | C18-C20 |
| Liver | C22 |
| Gallbladder | C23-C24 |
| Pancreas | C25 |
| Lung | C33-C34 |
| Melanoma of skin | C43 |
| Mesothelioma | C45 |
| Breast | C50 |
| Corpus uteri | C54-55 |
| Ovary | C56 |
| Prostate | C61 |
| Kidney | C64-C65 |
| Bladder | C67 |
| Brain, central nervous system | C70-72 |
| Thyroid | C73 |
| Non Hodgkin lymphoma | C82-C86, C96 |
| Multiple myeloma | C88-C90 |
| Leukaemia | C91-C95 |
| Abbreviation: ICD-10, the International Classification of Diseases, tenth revision. | |

| **Table S2. Characteristics of energy-adjusted carbohydrates intake in the study population** | | | | |
| --- | --- | --- | --- | --- |
|  | **Overall** | **Incident cancer events ^a^** | **No cancer participants** | ***P*** |
| Energy (kJ/d), median (IQR) | 8368.53 (7013.27, 9903.94) | 8498.06 (7113.99, 10018.85) | 8352.03 (7003.45, 9889.80) | <0.001 |
| Protein (g/d) ^b^, median (IQR) | 79.43 (69.55, 89.46) | 79.32 (69.82, 89.26) | 79.44 (69.51, 89.48) | 0.936 |
| Fat (g/d) ^b^, median (IQR) | 72.04 (63.10, 81.15) | 71.87 (62.97, 81.02) | 72.06 (63.11, 81.17) | 0.054 |
| Total carbohydrate (g/d) ^b^, median (IQR) | 253.68 (227.50, 278.22) | 253.13 (226.83, 277.83) | 253.74 (227.58, 278.24) | 0.008 |
| Starch (g/d) ^b^, median (IQR) | 127.50 (107.60, 147.64) | 126.33 (106.71, 146.54) | 127.63 (107.69, 147.77) | <0.001 |
| Englyst fiber (g/d) ^b^, median (IQR) | 17.27 (14.09, 20.81) | 17.30 (14.10, 20.78) | 17.27 (14.09, 20.81) | 0.634 |
| Total sugars (g/d) ^b^, median (IQR) | 121.64 (99.70, 145.29) | 122.00 (99.81, 145.80) | 121.60 (99.69, 145.23) | 0.154 |
| Free sugars (g/d) ^b^, median (IQR) | 56.69 (41.53, 74.53) | 56.83 (41.69, 75.01) | 56.67 (41.51, 74.48) | 0.219 |
| Non-free sugars (g/d) ^b^, median (IQR) | 60.48 (43.60, 79.74) | 60.65 (44.13, 79.76) | 60.46 (43.54, 79.73) | 0.202 |
| Fructose (g/d) ^b^, median (IQR) | 26.52 (18.76, 35.43) | 26.71 (18.83, 35.33) | 26.51 (18.76, 35.44) | 0.474 |
| Glucose (g/d) ^b^, median (IQR) | 24.92 (18.47, 32.37) | 25.03 (18.52, 32.35) | 24.91 (18.47, 32.37) | 0.276 |
| Lactose (g/d) ^b^, median (IQR) | 13.29 (8.88, 18.01) | 13.39 (9.01, 18.08) | 13.28 (8.86, 18.00) | 0.037 |
| Maltose (g/d) ^b^, median (IQR) | 5.40 (3.59, 7.61) | 5.36 (3.51, 7.64) | 5.40 (3.60, 7.61) | 0.096 |
| Sucrose (g/d) ^b^, median (IQR) | 44.53 (33.98, 56.82) | 44.64 (33.84, 57.19) | 44.52 (33.99, 56.77) | 0.545 |
| Other sugars (g/d) ^b^, median (IQR) | 1.73 (0.86, 2.91) | 1.67 (0.82, 2.85) | 1.74 (0.86, 2.92) | <0.001 |
| Abbreviation: IQR, interquartile range. |  |  |  |  |
| ^a^ Incident cancer events refer to any cancers that occurred during the follow-up period (ICD-10 codes: C00-C97, excluding C44). | | | | |
| ^b^ All carbohydrates were adjusted for total energy intake. | | | | |

| **Table S3. Hazard ratios for the associations between dietary carbohydrates across quartiles and the risk of overall cancer and various types of cancers** | | | | |
| --- | --- | --- | --- | --- |
|  | **Cases/total person-years** | **HR (95% CI)^b^** | ***P*** | ***P _trend_*** |
| **Total carbohydrates (g/d) ^a^** |  |  |  |  |
| **Non-Hodgkin lymphoma** |  |  |  |  |
| Q1 | 219/621,800 | Ref. |  | 0.002 |
| Q2 | 208/623,004 | 0.98 (0.80, 1.18) | 0.801 |  |
| Q3 | 255/623,441 | 1.22 (1.00, 1.47) | 0.047 |  |
| Q4 | 275/620,321 | 1.36 (1.10, 1.68) | 0.005 |  |
| **Esophageal cancer** |  |  |  |  |
| Q1 | 107/622,751 | Ref. |  | 0.018 |
| Q2 | 91/623,968 | 0.94 (0.71, 1.25) | 0.692 |  |
| Q3 | 74/624,579 | 0.78 (0.57, 1.06) | 0.109 |  |
| Q4 | 71/621,558 | 0.69 (0.49, 0.96) | 0.029 |  |
| **Starch (g/d) ^a^** |  |  |  |  |
| **Mesothelioma cancer** |  |  |  |  |
| Q1 | 27/621,333 | Ref. |  | 0.013 |
| Q2 | 27/624,371 | 1.06 (0.62, 1.82) | 0.819 |  |
| Q3 | 25/625,183 | 1.01 (0.58, 1.74) | 0.986 |  |
| Q4 | 46/622,784 | 1.84 (1.13, 3.01) | 0.014 |  |
| **Englyst fiber (g/d) ^a^** |  |  |  |  |
| **Colorectal cancer** |  |  |  |  |
| Q1 | 616/617,779 | Ref. |  | 0.001 |
| Q2 | 567/620,922 | 0.89 (0.79, 1.00) | 0.049 |  |
| Q3 | 613/621,810 | 0.94 (0.84, 1.05) | 0.273 |  |
| Q4 | 536/621,326 | 0.80 (0.71, 0.91) | <0.001 |  |
| **Kidney cancer** |  |  |  |  |
| Q1 | 180/619,977 | Ref. |  | 0.002 |
| Q2 | 162/623,214 | 0.95 (0.77, 1.18) | 0.673 |  |
| Q3 | 168/624,126 | 1.01 (0.81, 1.25) | 0.929 |  |
| Q4 | 105/623,622 | 0.64 (0.50, 0.82) | <0.001 |  |
| **Lung cancer** |  |  |  |  |
| Q1 | 388/619,873 | Ref. |  | <0.001 |
| Q2 | 286/623,271 | 0.82 (0.70, 0.96) | 0.012 |  |
| Q3 | 235/624,441 | 0.70 (0.59, 0.83) | <0.001 |  |
| Q4 | 252/623,510 | 0.74 (0.63, 0.88) | <0.001 |  |
| **Esophageal cancer** |  |  |  |  |
| Q1 | 123/620,397 | Ref. |  | 0.002 |
| Q2 | 84/623,700 | 0.76 (0.57, 1.01) | 0.055 |  |
| Q3 | 68/624,828 | 0.63 (0.47, 0.86) | 0.003 |  |
| Q4 | 68/623,932 | 0.64 (0.47, 0.87) | 0.004 |  |
| **Overall cancer** |  |  |  |  |
| Q1 | 4983/595,009 | Ref. |  | 0.007 |
| Q2 | 4960/596,790 | 0.97 (0.93, 1.00) | 0.081 |  |
| Q3 | 5070/596,655 | 0.97 (0.93, 1.01) | 0.129 |  |
| Q4 | 4977/596,493 | 0.94 (0.90, 0.98) | 0.004 |  |
| **Total sugars (g/d) ^a^** |  |  |  |  |
| **Non-Hodgkin lymphoma** |  |  |  |  |
| Q1 | 199/622,261 | Ref. |  | <0.001 |
| Q2 | 226/623,457 | 1.11 (0.91, 1.34) | 0.304 |  |
| Q3 | 233/622,942 | 1.13 (0.93, 1.37) | 0.229 |  |
| Q4 | 299/619,905 | 1.44 (1.18, 1.75) | <0.001 |  |
| **Free sugars (g/d) ^a^** |  |  |  |  |
| **Kidney cancer** |  |  |  |  |
| Q1 | 140/623,305 | Ref. |  | 0.011 |
| Q2 | 145/624,034 | 1.12 (0.88, 1.41) | 0.358 |  |
| Q3 | 149/623,862 | 1.15 (0.91, 1.45) | 0.255 |  |
| Q4 | 181/619,738 | 1.36 (1.07, 1.72) | 0.012 |  |
| **Lung cancer** |  |  |  |  |
| Q1 | 278/623,305 | Ref. |  | <0.001 |
| Q2 | 245/624,144 | 0.95 (0.79, 1.12) | 0.525 |  |
| Q3 | 285/623,850 | 1.12 (0.95, 1.33) | 0.192 |  |
| Q4 | 353/619,796 | 1.28 (1.08, 1.52) | 0.004 |  |
| **Non-free sugars (g/d) ^a^** |  |  |  |  |
| **Colorectal cancer** |  |  |  |  |
| Q1 | 621/618,757 | Ref. |  | <0.001 |
| Q2 | 578/621,142 | 0.88 (0.79, 0.99) | 0.031 |  |
| Q3 | 573/621,315 | 0.84 (0.75, 0.95) | 0.004 |  |
| Q4 | 560/620,623 | 0.80 (0.71, 0.91) | <0.001 |  |
| **Lung cancer** |  |  |  |  |
| Q1 | 370/620,878 | Ref. |  | <0.001 |
| Q2 | 305/623,431 | 0.90 (0.77, 1.05) | 0.177 |  |
| Q3 | 240/623,806 | 0.72 (0.60, 0.85) | <0.001 |  |
| Q4 | 246/622,981 | 0.74 (0.62, 0.89) | <0.001 |  |
| **Overall cancer** |  |  |  |  |
| Q1 | 4847/596,606 | Ref. |  | 0.005 |
| Q2 | 5088/596,424 | 1.01 (0.97, 1.05) | 0.701 |  |
| Q3 | 5050/596,007 | 0.97 (0.93, 1.01) | 0.160 |  |
| Q4 | 5005/595,910 | 0.95 (0.91, 0.99) | 0.020 |  |
| **Fructose (g/d) ^a^** |  |  |  |  |
| **Lung cancer** |  |  |  |  |
| Q1 | 405/620,439 | Ref. |  | <0.001 |
| Q2 | 280/623,599 | 0.79 (0.68, 0.92) | 0.003 |  |
| Q3 | 246/624,410 | 0.71 (0.60, 0.84) | <0.001 |  |
| Q4 | 230/622,648 | 0.68 (0.57, 0.81) | <0.001 |  |
| **Glucose (g/d) ^a^** |  |  |  |  |
| **Lung cancer** |  |  |  |  |
| Q1 | 394/620,628 | Ref. |  | <0.001 |
| Q2 | 261/623,679 | 0.75 (0.63, 0.87) | <0.001 |  |
| Q3 | 272/624,326 | 0.79 (0.67, 0.93) | 0.004 |  |
| Q4 | 234/622,462 | 0.68 (0.57, 0.82) | <0.001 |  |
| **Lactose (g/d) ^a^** |  |  |  |  |
| **Colorectal cancer** |  |  |  |  |
| Q1 | 636/619,513 | Ref. |  | <0.001 |
| Q2 | 581/620,689 | 0.89 (0.79, 0.99) | 0.035 |  |
| Q3 | 563/621,233 | 0.85 (0.76, 0.95) | 0.005 |  |
| Q4 | 552/620,403 | 0.81 (0.72, 0.92) | <0.001 |  |
| **Maltose (g/d) ^a^** |  |  |  |  |
| **Lung cancer** |  |  |  |  |
| Q1 | 280/624,027 | Ref. |  | 0.054 |
| Q2 | 259/624,843 | 0.95 (0.80, 1.12) | 0.541 |  |
| Q3 | 282/622,996 | 1.03 (0.87, 1.22) | 0.719 |  |
| Q4 | 340/619,230 | 1.14 (0.97, 1.35) | 0.113 |  |
| **Prostate cancer ^c^** |  |  |  |  |
| Q1 | 1208/243,861 | Ref. |  | 0.005 |
| Q2 | 979/209,822 | 0.92 (0.84, 1.00) | 0.044 |  |
| Q3 | 1061/228,023 | 0.95 (0.87, 1.03) | 0.196 |  |
| Q4 | 1662/410,771 | 0.89 (0.82, 0.96) | 0.002 |  |
| **Sucrose (g/d) ^a^** |  |  |  |  |
| **Lung cancer** |  |  |  |  |
| Q1 | 288/622,894 | Ref. |  | 0.002 |
| Q2 | 247/624,244 | 0.92 (0.78, 1.09) | 0.346 |  |
| Q3 | 256/623,653 | 0.99 (0.83, 1.17) | 0.903 |  |
| Q4 | 370/620,305 | 1.24 (1.06, 1.46) | 0.008 |  |
| **Non-Hodgkin lymphoma** |  |  |  |  |
| Q1 | 189/622,445 | Ref. |  | <0.001 |
| Q2 | 222/623,647 | 1.18 (0.97, 1.43) | 0.101 |  |
| Q3 | 268/622,796 | 1.43 (1.18, 1.73) | <0.001 |  |
| Q4 | 278/619,678 | 1.50 (1.24, 1.82) | <0.001 |  |
| Abbreviation: BMI, body mass index, CI, confidence interval, HR, hazard ratio, IQR, interquartile range, Ref., Reference, TDI, Thomson deprivation index. | | | | |
| ^a^ All carbohydrates were adjusted for total energy intake. | | | | |
| ^b^ Model was adjusted for age, sex, ethnicity, TDI, education, smoking status, alcohol drinking status, BMI, physical activity, energy-adjusted protein intake, and energy-adjusted fat intake. | | | | |
| ^c^ Associations between dietary carbohydrates and prostate cancer were analyzed only in men. | | | | |

| **Table S4. Sensitivity analyses on the associations between dietary carbohydrates and the risk of overall cancer and various types of cancers** | | | |
| --- | --- | --- | --- |
| **Energy-adjusted dietary carbohydrates per IQR increase** | **Cases/total person-years** | **HR (95% CI)** | ***P*** |
| ***Eliminating those who were diagnosed with any cancer (ICD-10 codes: C00-C97, excluding C44) within the first two years of follow-up ^b^*** | | | |
| **Total carbohydrates (g/d) ^a^** | | | |
| Non-Hodgkin lymphoma | 812/2,462,030 | 1.20 (1.07, 1.33) | 0.001 |
| Esophageal cancer | 311/2,465,120 | 0.80 (0.69, 0.92) | 0.002 |
| **Starch (g/d) ^a^** | | | |
| Mesothelioma cancer | 112/2,465,736 | 1.31 (1.05, 1.63) | 0.018 |
| **Englyst fiber (g/d) ^a^** | | | |
| Colorectal cancer | 2025/2,456,644 | 0.92 (0.87, 0.98) | 0.007 |
| Kidney cancer | 545/2,463,534 | 0.82 (0.74, 0.92) | <0.001 |
| Lung cancer | 1068/2,463,484 | 0.87 (0.80, 0.94) | <0.001 |
| Esophageal cancer | 311/2,465,120 | 0.83 (0.71, 0.96) | 0.011 |
| Overall cancer | 17492/2,382,395 | 0.97 (0.95, 0.99) | <0.001 |
| **Total sugars (g/d) ^a^** | | | |
| Non-Hodgkin lymphoma | 812/2,462,030 | 1.17 (1.07, 1.28) | <0.001 |
| **Free sugars (g/d) ^a^** | | | |
| Kidney cancer | 545/2,463,534 | 1.16 (1.05, 1.27) | 0.004 |
| Lung cancer | 1068/2,463,484 | 1.12 (1.05, 1.20) | <0.001 |
| **Non-free sugars (g/d) ^a^** | | | |
| Colorectal cancer | 2025/2,456,644 | 0.87 (0.82, 0.93) | <0.001 |
| Lung cancer | 1068/2,463,484 | 0.86 (0.79, 0.94) | <0.001 |
| Overall cancer | 17492/2,382,395 | 0.96 (0.95, 0.98) | <0.001 |
| **Fructose (g/d) ^a^** | | | |
| Lung cancer | 1068/2,463,484 | 0.84 (0.77, 0.91) | <0.001 |
| **Glucose (g/d) ^a^** | | | |
| Lung cancer | 1068/2,463,484 | 0.87 (0.80, 0.95) | 0.001 |
| **Lactose (g/d) ^a^** | | | |
| Colorectal cancer | 2025/2,456,644 | 0.91 (0.86, 0.96) | <0.001 |
| **Maltose (g/d) ^a^** | | | |
| Lung cancer | 1068/2,463,484 | 1.05 (1.01, 1.09) | 0.010 |
| Prostate cancer | 4281/1,085,727 | 0.98 (0.96, 1.00) | 0.044 |
| **Sucrose (g/d) ^a^** | | | |
| Lung cancer | 1068/2,463,484 | 1.11 (1.04, 1.18) | <0.001 |
| Non-Hodgkin lymphoma | 812/2,462,030 | 1.14 (1.05, 1.23) | <0.001 |
| ***Limiting participants to those with typical diet throughout 24-hour dietary evaluations ^b^*** | | | |
| **Total carbohydrates (g/d) ^a^** | | | |
| Non-Hodgkin lymphoma | 863/2,276,809 | 1.14 (1.03, 1.27) | 0.015 |
| Esophageal cancer | 315/2,280,690 | 0.83 (0.72, 0.96) | 0.014 |
| **Starch (g/d) ^a^** | | | |
| Mesothelioma cancer | 116/2,281,449 | 1.24 (0.99, 1.55) | 0.056 |
| **Englyst fiber (g/d) ^a^** | | | |
| Colorectal cancer | 2154/2,270,550 | 0.91 (0.86, 0.97) | 0.002 |
| Kidney cancer | 564/2,278,945 | 0.83 (0.75, 0.93) | 0.001 |
| Lung cancer | 1073/2,279,076 | 0.88 (0.81, 0.95) | 0.002 |
| Esophageal cancer | 315/2,280,690 | 0.82 (0.70, 0.95) | 0.007 |
| Overall cancer | 18394/2,181,330 | 0.97 (0.95, 0.99) | 0.004 |
| **Total sugars (g/d) ^a^** | | | |
| Non-Hodgkin lymphoma | 863/2,276,809 | 1.15 (1.05, 1.26) | 0.003 |
| **Free sugars (g/d) ^a^** | | | |
| Kidney cancer | 564/2,278,945 | 1.13 (1.02, 1.24) | 0.014 |
| Lung cancer | 1073/2,279,076 | 1.11 (1.03, 1.19) | 0.003 |
| **Non-free sugars (g/d) ^a^** | | | |
| Colorectal cancer | 2154/2,270,550 | 0.88 (0.83, 0.94) | <0.001 |
| Lung cancer | 1073/2,279,076 | 0.86 (0.79, 0.93) | <0.001 |
| Overall cancer | 18394/2,181,330 | 0.97 (0.95, 0.99) | 0.001 |
| **Fructose (g/d) ^a^** | | | |
| Lung cancer | 1073/2,279,076 | 0.84 (0.77, 0.92) | <0.001 |
| **Glucose (g/d) ^a^** | | | |
| Lung cancer | 1073/2,279,076 | 0.86 (0.79, 0.94) | <0.001 |
| **Lactose (g/d) ^a^** | | | |
| Colorectal cancer | 2154/2,270,550 | 0.91 (0.86, 0.96) | <0.001 |
| **Maltose (g/d) ^a^** | | | |
| Lung cancer | 1073/2,279,076 | 1.05 (1.02, 1.09) | 0.003 |
| Prostate cancer | 4572/1,002,365 | 0.98 (0.96, 0.99) | 0.004 |
| **Sucrose (g/d) ^a^** | | | |
| Lung cancer | 1073/2,279,076 | 1.09 (1.02, 1.15) | 0.008 |
| Non-Hodgkin lymphoma | 863/2,276,809 | 1.12 (1.04, 1.21) | 0.002 |
| ***Executing multiple imputations for missing covariates using the mice R package ^b^*** | | | |
| **Total carbohydrates (g/d) ^a^** | | | |
| Non-Hodgkin lymphoma | 982/2,549,525 | 1.18 (1.06, 1.30) | 0.001 |
| Esophageal cancer | 351/2,553,926 | 0.82 (0.72, 0.95) | 0.006 |
| **Starch (g/d) ^a^** | | | |
| Mesothelioma cancer | 127/2,554,756 | 1.39 (1.13, 1.71) | 0.002 |
| **Englyst fiber (g/d) ^a^** | | | |
| Colorectal cancer | 2387/2,542,635 | 0.91 (0.86, 0.96) | <0.001 |
| Kidney cancer | 636/2,551,911 | 0.84 (0.75, 0.93) | <0.001 |
| Lung cancer | 1209/2,552,039 | 0.88 (0.81, 0.94) | <0.001 |
| Esophageal cancer | 351/2,553,926 | 0.78 (0.67, 0.89) | <0.001 |
| Overall cancer | 20525/2,443,187 | 0.97 (0.96, 0.99) | 0.003 |
| **Total sugars (g/d) ^a^** | | | |
| Non-Hodgkin lymphoma | 982/2,549,525 | 1.19 (1.09, 1.29) | <0.001 |
| **Free sugars (g/d) ^a^** | | | |
| Kidney cancer | 636/2,551,911 | 1.15 (1.05, 1.25) | 0.003 |
| Lung cancer | 1209/2,552,039 | 1.11 (1.04, 1.18) | 0.001 |
| **Non-free sugars (g/d) ^a^** | | | |
| Colorectal cancer | 2387/2,542,635 | 0.88 (0.84, 0.94) | <0.001 |
| Lung cancer | 1209/2,552,039 | 0.86 (0.80, 0.93) | <0.001 |
| Overall cancer | 20525/2,443,187 | 0.97 (0.95, 0.99) | 0.001 |
| **Fructose (g/d) ^a^** | | | |
| Lung cancer | 1209/2,552,039 | 0.83 (0.77, 0.91) | <0.001 |
| **Glucose (g/d) ^a^** | | | |
| Lung cancer | 1209/2,552,039 | 0.86 (0.79, 0.93) | <0.001 |
| **Lactose (g/d) ^a^** | | | |
| Colorectal cancer | 2387/2,542,635 | 0.92 (0.87, 0.97) | 0.001 |
| **Maltose (g/d) ^a^** | | | |
| Lung cancer | 1209/2,552,039 | 1.06 (1.02, 1.09) | 0.002 |
| Prostate cancer | 5036/1,117,269 | 0.97 (0.96, 0.99) | 0.002 |
| **Sucrose (g/d) ^a^** | | | |
| Lung cancer | 1209/2,552,039 | 1.09 (1.03, 1.16) | 0.002 |
| Non-Hodgkin lymphoma | 982/2,549,525 | 1.15 (1.07, 1.23) | <0.001 |
| ***Additionally adjusted for prevalent hypertension, prevalent diabetes, and prevalent dyslipidemia ^c^*** | | | |
| **Total carbohydrates (g/d) ^a^** | | | |
| Non-Hodgkin lymphoma | 957/2,488,566 | 1.18 (1.07, 1.31) | <0.001 |
| Esophageal cancer | 343/2,492,857 | 0.82 (0.71, 0.94) | 0.004 |
| **Starch (g/d) ^a^** | | | |
| Mesothelioma cancer | 125/2,493,671 | 1.40 (1.14, 1.72) | 0.002 |
| **Englyst fiber (g/d) ^a^** | | | |
| Colorectal cancer | 2332/2,481,837 | 0.91 (0.87, 0.97) | 0.001 |
| Kidney cancer | 615/2,490,939 | 0.84 (0.76, 0.94) | 0.002 |
| Lung cancer | 1161/2,491,096 | 0.87 (0.80, 0.94) | <0.001 |
| Esophageal cancer | 343/2,492,857 | 0.79 (0.68, 0.91) | 0.001 |
| Overall cancer | 19990/2,384,947 | 0.97 (0.96, 0.99) | 0.003 |
| **Total sugars (g/d) ^a^** | | | |
| Non-Hodgkin lymphoma | 957/2,488,566 | 1.18 (1.08, 1.28) | <0.001 |
| **Free sugars (g/d) ^a^** | | | |
| Kidney cancer | 615/2,490,939 | 1.15 (1.05, 1.26) | 0.003 |
| Lung cancer | 1161/2,491,096 | 1.12 (1.05, 1.20) | <0.001 |
| **Non-free sugars (g/d) ^a^** | | | |
| Colorectal cancer | 2332/2,481,837 | 0.89 (0.84, 0.94) | <0.001 |
| Lung cancer | 1161/2,491,096 | 0.86 (0.79, 0.93) | <0.001 |
| Overall cancer | 19990/2,384,947 | 0.97 (0.95, 0.99) | 0.002 |
| **Fructose (g/d) ^a^** | | | |
| Lung cancer | 1161/2,491,096 | 0.84 (0.77, 0.91) | <0.001 |
| **Glucose (g/d) ^a^** | | | |
| Lung cancer | 1161/2,491,096 | 0.86 (0.80, 0.94) | <0.001 |
| **Lactose (g/d) ^a^** | | | |
| Colorectal cancer | 2332/2,481,837 | 0.91 (0.87, 0.96) | <0.001 |
| **Maltose (g/d) ^a^** | | | |
| Lung cancer | 1161/2,491,096 | 1.05 (1.02, 1.09) | 0.004 |
| Prostate cancer | 4910/1,092,477 | 0.97 (0.96, 0.99) | 0.001 |
| **Sucrose (g/d) ^a^** | | | |
| Lung cancer | 1161/2,491,096 | 1.10 (1.04, 1.17) | <0.001 |
| Non-Hodgkin lymphoma | 957/2,488,566 | 1.15 (1.07, 1.23) | <0.001 |
| ***Additionally adjusted for overall fruit, vegetable, and processed meats intake ^d^*** | | | |
| **Total carbohydrates (g/d) ^a^** | | | |
| Non-Hodgkin lymphoma | 957/2,486,507 | 1.18 (1.07, 1.31) | 0.001 |
| Oesophagus cancer | 343/2,490,798 | 0.83 (0.72, 0.95) | 0.008 |
| **Starch (g/d) ^a^** | | | |
| Mesothelioma cancer | 125/2,491,612 | 1.40 (1.14, 1.72) | 0.002 |
| **Englyst fiber (g/d) ^a^** | | | |
| Colorectal cancer | 2329/2,479,788 | 0.93 (0.87, 0.98) | 0.007 |
| Kidney cancer | 615/2,488,880 | 0.87 (0.78, 0.97) | 0.015 |
| Lung cancer | 1160/2,489,037 | 0.90 (0.83, 0.98) | 0.013 |
| Oesophagus cancer | 343/2,490,798 | 0.81 (0.70, 0.94) | 0.006 |
| Overall cancer | 19977/2,382,954 | 0.99 (0.97, 1.01) | 0.270 |
| **Total sugars (g/d) ^a^** | | | |
| Non-Hodgkin lymphoma | 957/2,486,507 | 1.18 (1.08, 1.29) | <0.001 |
| **Free sugars (g/d) ^a^** | | | |
| Kidney cancer | 615/2,488,880 | 1.14 (1.03, 1.25) | 0.008 |
| Lung cancer | 1160/2,489,037 | 1.10 (1.03, 1.17) | 0.006 |
| **Non-free sugars (g/d) ^a^** | | | |
| Colorectal cancer | 2329/2,479,788 | 0.89 (0.84, 0.95) | <0.001 |
| Lung cancer | 1160/2,489,037 | 0.91 (0.83, 1.00) | 0.041 |
| Overall cancer | 19977/2,382,954 | 0.99 (0.97, 1.01) | 0.248 |
| **Fructose (g/d) ^a^** | | | |
| Lung cancer | 1160/2,489,037 | 0.88 (0.81, 0.96) | 0.006 |
| **Glucose (g/d) ^a^** | | | |
| Lung cancer | 1160/2,489,037 | 0.91 (0.83, 0.99) | 0.029 |
| **Lactose (g/d) ^a^** | | | |
| Colorectal cancer | 2329/2,479,788 | 0.91 (0.86, 0.96) | <0.001 |
| **Maltose (g/d) ^a^** | | | |
| Lung cancer | 1160/2,489,037 | 1.04 (1.01, 1.08) | 0.018 |
| Prostate cancer | 4908/1,091,498 | 0.97 (0.95, 0.99) | <0.001 |
| **Sucrose (g/d) ^a^** | | | |
| Lung cancer | 1160/2,489,037 | 1.09 (1.03, 1.16) | 0.002 |
| Non-Hodgkin lymphoma | 957/2,486,507 | 1.15 (1.07, 1.23) | <0.001 |
| Abbreviation: BMI, body mass index, CI, confidence interval, HR, hazard ratio, IQR, interquartile range, TDI, Thomson deprivation index. | | | |
| ^a^ All carbohydrates were adjusted for total energy intake. | | | |
| ^b^ Model was adjusted for age, sex, ethnicity, TDI, education, smoking status, alcohol drinking status, BMI, physical activity, energy-adjusted protein intake, and energy-adjusted fat intake. | | | |
| ^c^ Model was adjusted for age, sex, ethnicity, TDI, education, smoking status, alcohol drinking status, BMI, physical activity, energy-adjusted protein intake, energy-adjusted fat intake, prevalent hypertension, prevalent diabetes, and prevalent dyslipidemia. | | | |
| ^d^ Model was adjusted for age, sex, ethnicity, TDI, education, smoking status, alcohol drinking status, BMI, physical activity, energy-adjusted protein intake, energy-adjusted fat intake, overall fruit, vegetable, and processed meats intake. | | | |
